# Gluten-Free Snacks Based on Brown Rice and Amaranth Flour with Incorporation of Cactus Pear Peel Powder: Physical, Nutritional, and Sensorial Properties

**DOI:** 10.1155/2018/7120327

**Published:** 2018-10-29

**Authors:** Dayanne Vigo Miranda, Meliza Lindsay Rojas, Sandra Pagador, Leslie Lescano, Jesús Sanchez-Gonzalez, Guillermo Linares

**Affiliations:** ^1^School of Agroindustrial Engineering, Universidad César Vallejo (UCV), AV. Víctor Larco Herrera 13009, Trujillo, Peru; ^2^Department of Agri-food Industry, Food and Nutrition (LAN), Luiz de Queiroz College of Agriculture (ESALQ), University of São Paulo (USP), AV. Padua dias 11, Piracicaba, SP, Brazil; ^3^School of Agroindustrial Engineering, Universidad Nacional de Trujillo (UNT), Av. Juan Pablo II s/n, Trujillo, Peru

## Abstract

An agroindustrial by-product (cactus pear peel) and whole grains flour (brown rice and amaranth) were used to present a gluten-free snack proposal. The effect of 5% (F1), 7% (F2), and 10% (F3) substitution of brown-rice flour for yellow cactus pear peel powder (*Opuntia ficus-indica*) on the snack physical, sensorial, and nutritional properties was evaluated. In addition, 20% of amaranth flour* (Amaranthus caudatus)* was used for all formulations. As the percentage of substitution increased, the a⁎ value increased, while the L⁎ decreased. The control snacks presented higher hardness, while the snacks with 10% substitution presented a greater crispness. The sensorial properties (overall liking, colour, crispness, and oiliness) reported that the samples containing cactus pear peel powder were the most accepted. The fat content decreased as the substitution percentage increased. The F3 formulation presented the best physical and sensorial properties and when compared with other commercial snack brands, it presented low fat and an adequate protein and fibre content. Therefore, snacks based on brown rice, amaranth, and cactus pear by-product could be considered as a good option of gluten-free product, contributing to reducing the lack of gluten-free products on the markets.

## 1. Introduction

The food industry has more and more challenges to meet current needs and consumer demands. On one hand, it offers healthy and safe foods with pleasant taste, appearance, and affordable cost. On the other hand, there is an environmental concern, where environment-friendly processes and agroindustrial by-products use are desired. Therefore, food scientists and engineers have the task of providing different alternatives considering the quality of the process, the product, and the special needs of the population, such as diseases that involve an immune response to any dietary compound, such as celiac disease.

Based on studies from United States [1] and Europe, the celiac disease incidence is about 1% of the world population; however, 85% to 90% of this has not been diagnosed nor treated [2]. The pathophysiology of celiac disease involves both the innate and adaptive immune response to dietary gluten. It is characterized by a permanent intolerance for gluten proteins present in dietary wheat, rye, and barley [3, 4]. It was reported that patients (about 50%) present atypical forms of celiac disease related to dermatitis herpetiformis, iron-deficiency anemia, neurologic problems, and others [5]. It was also reported that gluten causes gastrointestinal symptoms in subjects without celiac disease [6], such as irritable bowel syndrome [7]. Therefore, despite the growing number of people with celiac disease and other gluten-sensitivity-related problems [8], there is still a lack of gluten-free products on the market.

Although a gluten-free diet is effective in most patients, many patients find it unsatisfactory. This diet can be burdensome and can limit the quality of life; it is expensive, with bread and pasta substitutes costing substantially more than their gluten-containing counterparts [8]. In fact, some products based on oat, starches, hydrocolloids, gums, pulses ingredients, and fibre [9–11] were used to produce gluten-free products. Therefore, a greater variety of gluten-free products with good physical, nutritional, and sensory characteristics are required, in addition to affordable prices.

One way to produce quality food products with low prices, besides reducing the environmental impact, is the use of agroindustrial by-products. In fruit processing, around 70% of the raw material, such as peel and seeds, is considered waste. Peels are promising fruit by‐products because of their high content of insoluble and dietary fibre, pectin, and fructooligosaccharides, as well as phenolic compounds, proteins, minerals, and vitamins. In addition to the favourable physical or nutritional properties, the use of agroindustrial by‐products as food additives or supplements has gained increasing interest because their recovery may be economically attractive [12–15].

In this work, the use of an agroindustrial by-product was proposed, that is, the use of cactus pear peels. Cactus pear production using conventional system is around 30-80 tonnes ha ^−1^ and intensive system 179-263 tonnes ha^−1^ [16]. Cactus pear peel is the major by-product that represents 38% of fruit weight, resulting from fresh consumption or juice production [17]. The cactus pear peels were used as fibre source; the fibre content of cactus pear peel powder varies from 39% [18] to 64% [12]. On the other hand, there is a growing interest in adding value to Andean crops, so amaranth was used, an Andean grain that presents more than 13% of protein [19]. Additionally, brown-rice flour as the flour basis was used. In fact, the rice flour is gaining popularity as the alternative of wheat flour [20]. Some of the main nutritional compounds of cactus pear peel powder, amaranth flour, and brown-rice flour are presented in Table 1.

Therefore, the objective of the present work was to evaluate the effect of brown-rice flour substitution (5%, 7%, and 10%) with yellow cactus pear peel powder on the physical (instrumental colour, texture, and moisture content), sensory (overall liking and attributes of colour, texture, and oiliness), and nutritional properties (fat, protein, and fibre content) of gluten-free snacks with addition of amaranth flour (20%).

## 2. Materials and Methods

### 2.1. Raw Materials

#### 2.1.1. Brown-Rice and Amaranth Flour

The brown rice and amaranth were free of impurities and had low moisture content (amaranth grain: 10.47%; brown rice: 8.21%). The whole grains of rice and amaranth were ground in an electric mill for grains and the resulting product was sieved in a mesh N° 140 (0.105 mm) until obtaining a fine flour. The obtained flour was stored in hermetic containers at room temperature.

#### 2.1.2. Yellow Cactus Pear Peel Powder

Fresh yellow cactus pears (commonly known in Peru as tuna) produced in Santiago de Chuco-La Libertad were obtained from a local market (Trujillo, Peru). The fruits without damage were washed and sanitized. The peels were removed using a knife and cut in pieces of 2 cm^2^. The peels presented a pH of 5.73 ± 0.12 and °Brix of 8.90 ± 0.10. Subsequently, the cut peels were dried at 70°C for 24 h using an oven (BOX 1720, MEMMERT, Germany) until reaching 1.84 g water/100g dry matter.

Dried peels were ground using the same grain electric mill and then sieved using a mesh N° 100 (0.149 mm) until obtaining a fine powder. The obtained powder was stored in hermetic containers at room temperature.

### 2.2. Formulation and Preparation of Snacks

The snacks were prepared according to the formulations shown in Table 2. The control snacks were prepared with 20% of amaranth flour and 80% of brown-rice flour. The amaranth flour percent (20%) was maintained constant for all formulations. Formulations 1, 2, and 3 were prepared by substitution of brown-rice flour with cactus pear peel powder. Substitutions were conducted based on 5%, 7%, and 10% of the weight of the brown-rice flour.

**Figure 1:**
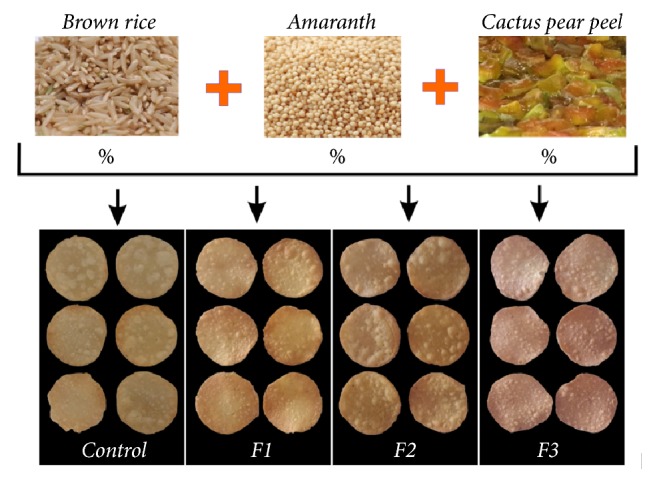
Snacks obtained with the different used formulations.

The snacks were prepared for each formulation in the same manner. Firstly, according to the type of formulation (Table 2), the cactus pear peel powder, the amaranth flour, and the brown-rice flour were mixed. For each 100 g of mixture, 5 g of salt, 70 mL of water, and 40 g of butter were added (this quantity provided a texture appropriate to the dough, which allowed future handling without rupture). The mixture was then kneaded to form a smooth dough. The dough was steam precooked for 20 min. Subsequently, the dough was cooled at room temperature, followed by sheeting until reaching a final thickness of 1 mm. The sheets of dough were then cut into circles using a mould of 4 cm diameter. Finally, they were fried in sunflower oil at 170°C for 5 s; the obtained snacks are shown in Figure 1. The snacks were stored in hermetic containers in a cool and dry place for posterior analyses. Four repetitions of each formulation were performed.

### 2.3. Physical Properties

#### 2.3.1. Instrumental Colour

The instrumental colour of fried snacks was measured on the snack surface according to the method described by Mir et al. [22], using a spectrophotometer (Konica Minolta CM-5, Japan). The CIE (*Commission Internationale d'Eclairage*) colour scale was used, where parameters of L^*∗*^ (lightness), a^*∗*^ (green to red), and b^*∗*^ (blue to yellow) were measured.

#### 2.3.2. Instrumental Texture

**Figure 2:**
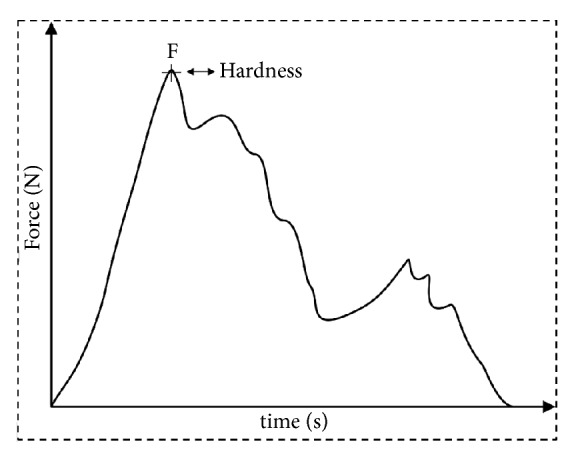
Typical curve of force (N) versus time (s) showing the maximum compression force used for describing the snack texture.

The instrumental texture of fried snacks was evaluated according to Alam et al. [23] by a compressive test using a texturometer (TA-HDplus, UK). A stainless-steel spherical probe (P/0.25 s) was inserted at a constant rate of 1 mm/s over a distance of 3 mm until it cracked the snack. The maximum compression force considered as the highest point of the force (N) versus time (s) curve was used for describing the sample texture (in terms of hardness) (Figure 2). The analysis was performed in triplicate for each formulation replicate, that is, 12 texture readings for each formulation.

### 2.4. Moisture Content

The moisture content of the raw materials and snacks was measured by drying the crushed snacks samples at 105°C using a moisture analyzer (MX-50, A&D Company, Tokyo, Japan); for this a HI (high) accuracy mode was selected. The moisture measurement was completed when the drying rate was lower than 0.02%/min.

### 2.5. Sensorial Properties

The snack sensorial properties were evaluated according to Ho and Abdul Latif [24] and Cruz et al.[25], with some modifications. A total of 97 untrained panellists (63% male and 37% female, age range: 18-25 years) were recruited for the sensory analysis. All participants were informed in an orientation session about the objectives and the use of the scale and signed an ethics consent form. The coded samples were provided to each panellist in a monadic sequential order. For each snack formulation, using a 7-point structured hedonic scale, consumers evaluated the overall liking, the colour (defined as the intensity of colour present on the surface of the snack; it varied from (1) very pale to (7) Very dark), the oiliness (defined as the taste and sensation of oil in snacks; it varied from (1) any oiliness to (7) very intense oiliness), and crispiness (defined as the degree of “crunch” or sound produced when the snack is bitten; it varied from (1) very slight sound to (7) very intense sound).

### 2.6. Nutritional Properties

#### 2.6.1. Fat Content

The fat percentage (%F) of the snacks was determined using the Soxhlet extraction method, AOAC Method N° 922.06 [26]. After frying, the samples were dried, triturated, and placed inside a thick filter paper thimble, which was placed into the Soxhlet equipment for fat extraction using a combination of analytical grade ethyl ether and petroleum ether (1:1) (Merck, Darmstadt). The determination was performed in triplicate for each formulation replicate.

After having carried out the physical, sensorial, and fat content analyses, the formulation that obtained the best properties and sensory acceptance was selected. For the snacks processed using this formulation, the amounts of protein and fibre were determined. In addition, the fat, protein, and fibre composition of the snacks made in this study was compared with those quantities reported for some brands of commercial snacks. The used brands were* Los cuates* (Karinto S.A.C, Lima, Peru),* Lay's ® classic*, and* Doritos nacho cheese flavoured* (Frito-Lay Inc., Texas, USA) from PepsiCo Inc. Company.

#### 2.6.2. Protein Content

The proteins were determined using the Kjeldahl digestion and distillation method, According to the methodology described by the AOAC method N° 920.87 [27].

#### 2.6.3. Crude Fibre Content

The crude fibre content was determined according to the methodology described by the AOAC method N° 978.10 [28].

### 2.7. Statistical Analysis

Data were analyzed using the Statgraphics Centurion (StatSoft, USA) software. Analyses were performed through analysis of variance (ANOVA) at 95% confidence (*p* < 0.05), followed by Duncan test to identify significant differences among treatments.

## 3. Results and Discussion

### 3.1. Physical Properties

#### 3.1.1. Instrumental Colour

In snacks, the colour is one of the most important attributes judged by the consumer. Visually, as shown in Figure 1, the snacks presented differences in colour; this was evidenced objectively through the instrumental measurement.  Table 3 shows the instrumental colour parameters value. The L*∗* parameter (lightness) decreased as the percentage of cactus peel powder increased. Therefore, less lightness was detected in formulations with the highest percentage of cactus pear peel powder (F3). Contrary to the L*∗* values, the a*∗* values increased as the percentage of brown-rice flour substitution increased, the highest a*∗* value being that for the F3 formulation. The values of b*∗* were kept similar in the F2 and F3 samples, with a slight increase in F1 when compared to the control. Lastly, the total colour difference (ΔE) increased as the percentage of cactus pear powder increased.

In Table 3, it is observed that the lightness value (L*∗*) decreased as the percentage of substitution of brown-rice flour increased. This result makes sense because the cactus pear peel powder used contain pigments, in addition to other reactions that occur with the temperature increase during frying. In fact, the betalain and chlorophyll are pigments of the cactus pear peel powder, which are susceptible to the temperature, pH, and light [29]. These pigments generate compounds of dark brown colouration that decreased the lightness. Additionally, previous studies attributed the changes in L*∗* to reducing sugars content present in the raw material. If this content is low, golden snacks will be obtained; however, if the content of reducing sugars is excessive, it will cause a deep brown colouration in the final product decreasing the lightness [30].

The a*∗* parameter (from green (-) to red (+)) is used to determine the optimal frying point of fried products. In this work, the value of a*∗* increased as the percentage of brown-rice flour substitution was increased (Table 3). That is, the colour of the samples was approximated to a red colour as the percentage of cactus pear powder increased. Meanwhile, in the case of b*∗* parameter (from blue (-) to yellow (+)), for all samples obtained similar and positive results, comparable results were reported by Heredia and Castelló [31]. The obtained results could be due to the used variety of cactus pear, where peel powder was yellow; therefore it conferred to the formulations a yellowish colour.

Finally, all variations in the L*∗*, a*∗*, and b*∗* parameters for each formulation impacted the total colour difference (ΔE) (Table 3). As stated above, both the raw material composition and the reactions that occur during processing contributed to this colour differences. For example, the different chemical reactions such as caramelization (Maillard reaction), nonenzymatic reactions, and structural changes were accelerated by the high temperatures during the frying process [32].

#### 3.1.2. Texture

The texture is another important sensory attribute for the fried snacks acceptance [33]. Usually, the instrumental texture in foods like snacks is determined through a compression test, where the force-displacement curves are obtained (Figure 2). The curves usually have an irregular appearance with several fracture events. This behaviour is observed in crispy foods [34]. The first important drop of force is associated with a major structural breakdown; in this region, the probe mainly deforms the sample [35]. The texture of the snacks was expressed as the maximum force needed to break the snack.

**Figure 3:**
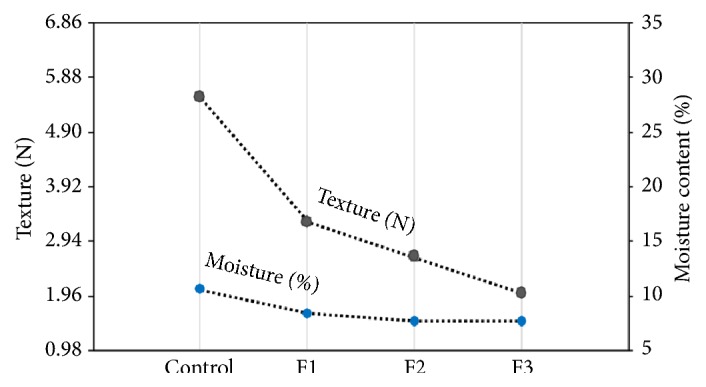
Texture (force (N)) and moisture content (%) for each snack formulation.

Significant differences (*p* < 0.05) among the texture of all the samples were observed. The force needed to break the snack decreases as the percentage of substitution of brown-rice flour increases (Figure 3). The control samples obtained the highest value (5.52 ± 0.03 N), while the F3 (10% substitution) samples obtained the lower value of force (2.02 ± 0.02 N). Additionally, it was observed that the moisture content (%) is directly proportional to the strength needed to break the snack (Figure 3).

According to Kita and Lisińska [36], samples with very low solid content and high fat content are less crisp and stickier. This occurred with the control samples, which showed high moisture (Figure 3) and fat content (Table 5). In addition, there are several factors influencing texture properties such as raw material composition, frying temperature, and oil type [37]. In this work, the temperature and time of frying and the oil type were the same; therefore, probably the observed differences on the snack properties were due to their different composition. For example, the control snacks contained a high quantity of brown-rice flour. The brown-rice flour, among others, contains fibre and starch. According to Pacheco-Delahaye [38], during frying, the starch contributes to the crust formation, causing a hard product. As observed, the control snacks presented the higher hardness value. In contrast, the F3 samples presented lower hardness results, indicating that they were more fragile or crisp.

### 3.2. Moisture Content

Figure 3 shows the moisture content (%) tendency obtained regarding each formulation. As observed, the force (N) and moisture content (%) showed direct dependency on the snack composition, which decreased as the percentage of brown-rice flour substitution increased. Therefore, the control snacks showed the greater hardness and moisture (%), while the F3 (10% cactus pear peel powder) showed the lowest moisture content with most crispy texture.

Different variables act simultaneously and influence the obtained results [39]. According to van Koerten and Schutyser [40] a lower moisture content results in an increased porosity. The increased porosity decreases the breaking resistance, which in turn results in a crispier behaviour after frying. This could have occurred when the percentage of brown-rice flour substitution increased, where the snacks porosity probably increased, resulting in the observed behaviour.

The observed moisture content could be attributed to the rice flour composition, which contributed to the crust formation. In other products that also contain starch, such as potato chips, the crust formation was reported [41]. Therefore, probably the snacks that contained more quantity of brown-rice flour (as in the case of control snacks) formed rapidly crust increasing the external resistances to water exit. Consequently, the water inside the snack remained trapped, increasing the moisture content.

### 3.3. Sensorial Properties

The analysis of variance (ANOVA) showed no significant effect on the consumers. However, there was a significant effect for the snacks formulation (*p* < 0.05).

**Figure 4:**
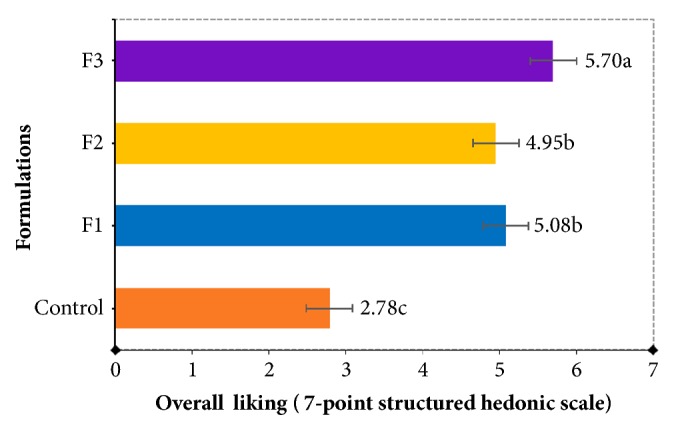
Average of the overall liking scores of each formulation, using a 7-point hedonic scale.

Figure 4 shows the overall liking for each snack formulation. The snacks that were most liked were those prepared using the formulations F1, F2, and F3; these snacks have an average acceptance of more than 5 on a hedonic scale of 7 points. Therefore, the three formulations mentioned above have a great possibility of being accepted by consumers. However, among these three formulations, the F3 formulation has the greatest acceptance. In contrast, the sample with the lowest acceptance by consumers was the control sample. Therefore, it can be inferred that the snacks without the addition of cactus pear peel powder presented low acceptance values.

Regarding the specific sensory attributes of snacks (colour, crispiness, and oiliness) (Table 4), the colour attribute scores were among “slightly pale” and “moderately dark” colour. It was observed that the colour score increased as the percentage of cactus pear peel powder increased. That is, the F3 formulation obtained the highest colour intensity punctuation; in fact, it was evidenced instrumentally where these samples showed higher a*∗* and lower L*∗* values. For the crispiness attribute, scores between 2.4 and 5.1 were obtained, which are equivalent in the hedonic scale to “mildly slight” and “slightly intense.” In this regard, the snacks prepared with the F3 formulation were the crispiest. Furthermore, significant differences were found in the oiliness attribute, where the snacks of the F3 formulation showed fewer scores, with an average score of 2.97. It means that these snacks presented less taste and an oily sensation. In contrast, the control sample obtained the highest oiliness score of 4.19, equivalent to “slightly intense.” The oiliness score agreed with what is reported in Table 5, where the F3 and control samples showed the lowest and highest fat percentage, respectively.

### 3.4. Nutritional Properties

#### 3.4.1. Fat Content (%)

The fat of fried snacks is an important component, which affects their flavour, texture, and shelf life. In the last years, the demand for healthy products, such as low-fat products, has been increasing [42]. Therefore, different studies were performed to obtain low-fat products, for example, by applying pretreatments [25, 43]. In this study, the use of 10% cactus pear peel powder (F3) allowed obtaining snacks with the lowest fat percentage, while the highest percentage of fat was obtained for control snacks (Table 5).

In addition to the fat content of the raw materials and the fat used in the elaboration of the dough, during deep fat frying, there are coupled heat and mass transfer phenomena. During the mass transfer moisture loss and oil uptake simultaneously occur [25].

The mass transfer (moisture loss and oil uptake) is affected by the food-related properties (such as structure, composition, porosity, vapour pressure, and surface roughness) and by the process conditions (such as temperature, frying time, and type of used oil) [36, 43–48]. In fried products, the highest percentage of oil absorbed is on the surface, where the structure of the formed crust is a key factor [41, 49].

In fact, the control snacks showed the greater hardness and moisture (%) (Figure 3) and a high fat content (Table 5), while F3 (10% cactus pear peel powder) showed the lowest fat and moisture content with most crispy texture. Therefore, the high fat and moisture content observed in the control samples could be attributed to the crust formation. The oil content of the crust was higher than that of the core regions [41]. Probably the control snacks that contained more quantity of brown-rice flour formed rapidly crust storing more oil, thus increasing %F.

#### 3.4.2. Protein and Fibre Content

Under the process conditions, F3 formulation allowed obtaining snacks with the best physical and sensory properties (low fat content, adequate colour and hardness, and greater overall acceptability). Therefore, for this formulation, analyses of proteins and fibre content were performed and compared with other brands typically found in markets (Table 6).

As shown in Table 6, the F3 sample presented lower fat content compared to the other brands; in addition, it contained a slightly higher percentage of proteins (7.22%). This can be due to the protein content of the used raw materials. However, apparently, it had a lower fibre content (2.13%); this is because the fibre content value in this work was reported as crude fibre and the value reported in the label of commercial snacks corresponds at the dietary fibre content. When the crude fibre method is used, the dietary fibre (DF) content is significantly underestimated, since a large part of the hemicellulose and lignin is dissolved, as well as varying amounts of cellulose and all the soluble fibres. The DF values are generally 3 to 5 times higher than the crude fibre [50, 51]. Therefore, the F3 formulation could present a high value of dietary fibre. Although not evidenced, this possibility is supported by the types of flours that were used, both rice and amaranth, which were of an integral type. Further, previous studies reported that cactus pear peel powder has a 64.15% total dietary fibre (Table 1): 33.48% insoluble and 30.67% soluble [12].

## 4. Conclusions

The substitution of brown-rice flour with cactus pear peel powder directly influenced the physical properties of the snacks. At a higher percentage of substitution, the a*∗* colour parameter value increased, while the lightness (L*∗*) decreased with smaller variations in the b*∗* value. In addition, the instrumental texture values decreased as the percentage of substitution increased; the control snacks presented higher hardness values, while the snacks with the higher percentage of substitution (such as F3) had lower hardness values, suggesting a greater crispness. The evaluation of sensorial properties reported that the samples containing cactus pear peel powder were the most accepted. For the specific sensory attributes of colour, texture, and oiliness, it was obtained that the higher percentage of substitution obtained better values of acceptance. The fat content decreased as the substitution percentage increased. Therefore, the F3 formulation presented the best physical and sensorial properties and low fat content. Additionally, when compared with other commercial brands, the F3 snacks elaborated in this study presented an adequate protein and fibre content. Therefore, the elaboration of snacks is a good proposal to exploit the use of agroindustrial by-products and provide gluten-free products.

## Figures and Tables

**Table 1:** Main nutritional compounds of cactus pear peel powder, amaranth flour, and brown rice flour.

Compound (x 100g of product)		Cactus pear peel powder ^(1)(2)^	Amaranth flour ^(3)^	Brown rice flour ^(3)^
Protein	g	5.71	13.33	7.23
Total Fat	g	3.33	6.67	2.78
Fibre, total dietary	g	64.15	11.10	4.60
Carbohydrates	g	71.84	66.67	76.48
Total carotenoids	mg	217.11	Nr	nr
Calcium	mg	Nr	178.00	11
Iron	mg	Nr	7.20	1.98

nr: nonreported. Mean values obtained from ^(1)^ Elhassaneen and Ragab [18], ^(2)^ Diaz‐Vela and Totosaus [12], and ^(3)^ USDA [21].

**Table 2:** Snacks formulation.

**Formulation **	**Cactus pear peel powder (**%**)**	**Amaranth flour (**%**)**	**Brown rice flour ** **(**%**)**
Control	-	20	80
Formulation 1 (F1)	5	20	75
Formulation 2 (F2)	7	20	73
Formulation 3 (F3)	10	20	70

**Table 3:** Instrumental colour parameters in the different formulations of snacks*∗*.

**Colour parameters**	**Formulations**			
	**Control**	**F1**	**F2 **	**F3 **
L*∗*	50.17 ± 2.22^a^	43.29 ± 1.24^b^	34.41 ± 0.29^b^	32.11 ± 0.53^c^
a*∗*	4.55 ± 0.82^a^	5.94 ± 0.93^a^	6.72 ± 0.42^a^	10.37 ± 0.95^b^
b*∗*	22.21 ± 1.59^a^	23.71 ± 0.64^b^	21.80 ± 0.25^a^	22.80 ± 0.73^a^
ΔE	-	7.35 ± 1.44^a^	15.96 ± 1.98^b^	19.10 ± 2.23^b^

*∗*mean ± standard deviation. Differences among letters indicate significant differences among formulations (*α*=5%).

**Table 4:** Results of the sensory attributes (colour, crispiness, and oiliness) evaluated for the different snacks formulation*∗*.

**Attributes**	**Formulation**			
	**Control**	**F1**	**F2**	**F3**
**Colour**	3.15 ± 1.12^a^	4.21 ± 1.31^b^	5.43 ± 0.89^c^	5.79 ± 0.82^d^
**Crispiness**	2.43 ± 1.29^a^	4.58 ± 1.26^b^	4.9 ± 1.10^c^	5.10 ± 1.29^c^
**Oiliness**	4.19 ± 1.47^a^	3.7 ± 1.23^b^	3.94 ± 1.31^b^	2.97 ± 1.19^c^

*∗*mean ± standard deviation. Differences among letters indicate significant differences among formulations (*α*=5%).

**Table 5:** Fat content (%) for the snacks obtained with different formulations*∗*.

**Formulations**	**Fat content (**%**)**
**Control**	31.75 ± 0.01^a^
**F1**	30.08 ± 0.01^b^
**F2**	30.41 ± 0.01^b^
**F3**	26.89 ± 0.13^c^

*∗*mean ± standard deviation. Differences among letters indicate significant differences among formulations (*α*=5%).

**Table 6:** Nutritional comparison of fried snacks with other brands.

**Compound**	**Composition (**%**)**			
	**F3**	**Los Cuates** **∗**	**Lay's® Classic** **∗**	**Doritos Nacho cheese flavoured** **∗**
**Fat**	26.89	36.73	35.71	28.57
**Protein**	7.22	7.14	7.14	7.14
**Fibre**	2.13 (CF)	3.57 (DF)	3.57 (DF)	3.57 (DF)

*∗*Data obtained from nutrition facts reported in the product label. CF: crude fibre; DF: dietary fibre.

## Data Availability

The experimental data used to support the findings of this study are available from the corresponding author upon request.
